# Supplementary material for: Analysis of rod/cone gap junctions from the reconstruction of mouse photoreceptor terminals
Source: eLife. 2022 Apr 26;11:e73039. doi: 10.7554/eLife.73039 (PMC9170248; doi:10.7554/eLife.73039)
Supplement: Transparent reporting form [file elife-73039-transrepform1.docx]

***eLife’s* transparent reporting form**

We encourage authors to provide detailed information *within their submission* to facilitate the interpretation and replication of experiments. Authors can upload supporting documentation to indicate the use of appropriate reporting guidelines for health-related research (see [EQUATOR Network](http://www.equator-network.org/%20)), life science research (see the [BioSharing Information Resource](https://biosharing.org/)), or the [ARRIVE guidelines](http://www.plosbiology.org/article/info:doi/10.1371/journal.pbio.1000412) for reporting work involving animal research. Where applicable, authors should refer to any relevant reporting standards documents in this form.

If you have any questions, please consult our Journal Policies and/or contact us: [editorial@elifesciences.org](mailto:editorial@elifesciences.org).

**Sample-size estimation**

- You should state whether an appropriate sample size was computed when the study was being designed
- You should state the statistical method of sample size computation and any required assumptions
- If no explicit power analysis was used, you should describe how you decided what sample (replicate) size (number) to use

Please outline where this information can be found within the submission (e.g., sections or figure legends), or explain why this information doesn’t apply to your submission:

Sample sizes are based on previous experiments in the lab and comparable to those used in published literature. Sample sizes for each experiment are reported in the figure legends and/or results section.

**Replicates**

- You should report how often each experiment was performed
- You should include a definition of biological versus technical replication
- The data obtained should be provided and sufficient information should be provided to indicate the number of independent biological and/or technical replicates
- If you encountered any outliers, you should describe how these were handled
- Criteria for exclusion/inclusion of data should be clearly stated
- High-throughput sequence data should be uploaded before submission, with a private link for reviewers provided (these are available from both GEO and ArrayExpress)

Please outline where this information can be found within the submission (e.g., sections or figure legends), or explain why this information doesn’t apply to your submission:

Number of biological replicates and technical replicates were decided from the experience, literature survey. For confocal and EM imaging several fields were imaged without bias and the representative figure has been used in Figure panels. All experiments were performed minimum 3 times for confocal and 2 times for EM analysis, and the details are mentioned in methods or in figure legends. Excluded data and criterion for removal is stated in the figure legend.

**Statistical reporting**

- Statistical analysis methods should be described and justified
- Raw data should be presented in figures whenever informative to do so (typically when N per group is less than 10)
- For each experiment, you should identify the statistical tests used, exact values of N, definitions of center, methods of multiple test correction, and dispersion and precision measures (e.g., mean, median, SD, SEM, confidence intervals; and, for the major substantive results, a measure of effect size (e.g., Pearson's r, Cohen's d)
- Report exact p-values wherever possible alongside the summary statistics and 95% confidence intervals. These should be reported for all key questions and not only when the p-value is less than 0.05.

Please outline where this information can be found within the submission (e.g., sections or figure legends), or explain why this information doesn’t apply to your submission:

Raw data is presented by scatter plot (and histogram if appropriate) in addition to box chart. Data is presented as mean±SD in manuscript and other statistical values (median, quartiles, min/max) are in the box chart. Exact statistical values are also provided in supplemental tables. Unpaired t-test is used to test the statistical difference between two experimental groups. Exact p-values are shown in supplemental tables.

(For large datasets, or papers with a very large number of statistical tests, you may upload a single table file with tests, Ns, etc., with reference to sections in the manuscript.)

**Group allocation**

- Indicate how samples were allocated into experimental groups (in the case of clinical studies, please specify allocation to treatment method); if randomization was used, please also state if restricted randomization was applied
- Indicate if masking was used during group allocation, data collection and/or data analysiss.

Please outline where this information can be found within the submission (e.g., sections or figure legends), or explain why this information doesn’t apply to your submission:

Groups were allocated according to position of cells (all 29 cone cells or central 13 cones) and cell-type (green or blue cone). These are explained in manuscript with biological and statistical reasons. Statistical values of the central 13 cones were validated to compare with larger group of 29 cones.

**Additional data files (“source data”)**

- We encourage you to upload relevant additional data files, such as numerical data that are represented as a graph in a figure, or as a summary table
- Where provided, these should be in the most useful format, and they can be uploaded as “Source data” files linked to a main figure or table
- Include model definition files including the full list of parameters used
- Include code used for data analysis (e.g., R, MatLab)
- Avoid stating that data files are “available upon request”

Please indicate the figures or tables for which source data files have been provided:

Source data used to create figures have been provided in the excel-row data for Figures 2, 3, 4, 5 and 8. Skelton data in Figure 4 and 5 have been provided as a file format for Knossos software (https://knossos.app/).
